# Evaluation of electric phrenic nerve stimulation patterns for mechanical ventilation: a pilot study

**DOI:** 10.1038/s41598-023-38316-1

**Published:** 2023-07-12

**Authors:** Arnhold Lohse, Philip von Platen, Carl-Friedrich Benner, Matthias Manfred Deininger, Teresa Gertrud Seemann, Dmitrij Ziles, Thomas Breuer, Steffen Leonhardt, Marian Walter

**Affiliations:** 1 Medical Information Technology, Faculty of Electrical Engineering and Information Technology, RWTH Aachen University, 52072 Aachen, Germany; 2 Department of Intensive and Intermediate Care, Medical Faculty, RWTH Aachen University, 52072 Aachen, Germany

**Keywords:** Peripheral nervous system, Preclinical research, Health care, Electrical and electronic engineering

## Abstract

Diaphragm atrophy is a common side effect of mechanical ventilation and results in prolonged weaning. Electric phrenic nerve stimulation presents a possibility to avoid diaphragm atrophy by keeping the diaphragm conditioned in sedated patients. There is a need of further investigation on how to set stimulation parameters to achieve sufficient ventilation. A prototype system is presented with a systematic evaluation for stimulation pattern adjustments. The main indicator for efficient stimulation was the tidal volume. The evaluation was performed in two pig models. As a major finding, the results for biphasic pulses were more consistent than for alternating pulses. The tidal volume increased for a range of pulse frequency and pulse width until reaching a plateau at 80–120 Hz and 0.15 ms. Furthermore, the generated tidal volume and the stimulation pulse frequency were significantly correlated (0.42–0.84, $p<0.001$). The results show which stimulation parameter combinations generate the highest tidal volume. We established a guideline on how to set stimulation parameters. The guideline is helpful for future clinical applications of phrenic nerve stimulation.

## Introduction

Mechanical ventilation is a respiratory support which up to ${36.4}\%$ of the patients in the intensive care units receive^1^. In sedated patients, the lung is ventilated by applied volume or pressure through the respirator while the patients’ diaphragm remains inactive, which leads to diaphragm atrophy. Diaphragm atrophy can occur within hours after the initial start of mechanical ventilation^2^. After treating the underlying cause of respiratory failure, clinicians typically perform ventilator weaning^3^. The process of weaning refers to the reduction of ventilatory support, aiming for the patient to breathe sufficiently to be extubated and thereafter breathe completely without a ventilator^4^. A critical prerequisite for this is, among other things, that the patient has adequate respiratory muscle strength. The diaphragm, the main respiratory muscle, is crucial in this regard. However, ${30}\%$ of the patients are difficult to wean and ${10}\%$ require prolonged weaning. These patients account for ${40}\%$ of total patient-days in the intensive care unit and become the most expensive in-house patients in hospitals^1^. We note that the weaning failure, resulting in prolonged mechanical ventilation, causes diaphragmatic atrophy^5^.

A possible alternative or support to mechanical ventilation is phrenic nerve stimulation (PNS) to enable the diaphragm to contract by nerve stimulation and thus avoid atrophy and deconditioning during ventilation. PNS is a technique in which a stimulator device provides electrical pulses to the phrenic nerve to invoke a contraction of the diaphragm. In an animal trial with pigs, it has recently been shown that electrical transvenous PNS reduces diaphragm weakness^6^. The transvenous PNS system was validated in humans afterwards^7^. In another trial, twenty-five pigs were ventilated successfully for more than 50 h with synchronous transvenous electrical PNS support on every mechanically ventilated breath or every second breath^8^. The PNS feasibility of implantable stimulators in a single human subject was evaluated^9^. Here, the study focus was to compare different stimulation devices and electrode positions. In one experiment the pulse charge was varied for a constant pulse frequency and in another one the pulse frequency was varied for a constant pulse charge. However, in these earlier studies, only a few different patterns of stimulation have been tested. A systematic investigation on electrode positioning and PNS parameters was performed for transcutaneously placed surface electrodes on the neck in humans^10^ recently. After the electrode position was fixed, the pulse amplitude and pulse width were optimized. Afterwards, the pulse frequency was adjusted. The humans were healthy and not sedated.

Our work described in this article aims to relate the PNS parameters, primarily the pulse frequency and pulse width, to the tidal volume. This paper shows how PNS pattern parameters can be evaluated and describes the results of the PNS pattern evaluation performed during two animal tests with sedated pigs. The results are used to give recommendations on how to optimally set PNS parameters regarding PNS effectivity.

## Methods

### Experiment overview

**Figure 1:**
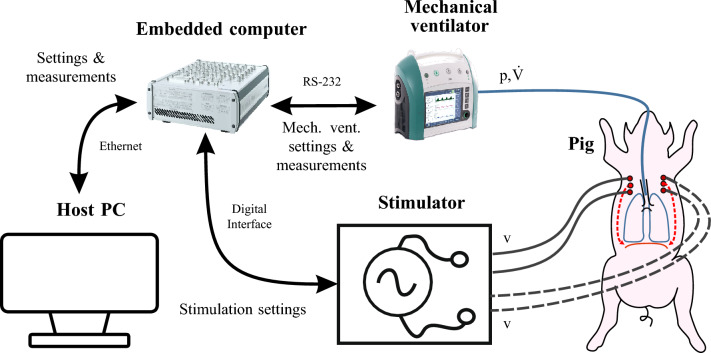
Setup of the animal test for PNS.

The experimental setup for PNS is shown in Fig. 1. The user operates a host PC running Matlab Simulink 2019b (The MathWorks Inc., Natick, USA) and dSPACE Control Desk 7.1 (dSPACE GmbH, Paderborn, Germany) to communicate with a real time embedded computer (MicroLabBox, dSPACE). The embedded computer controls the stimulator described in Section “Stimulator design” via the serial peripheral interface(SPI) and digital outputs. The stimulator is connected to stimulation cannulas (SonoPlex STIM, PAJUNK Medical Produkte GmbH, Geisingen, Germany) which were coated with an isolation lacquer before the experiments and percutaneously placed near the left and right phrenic nerve of the healthy subject pigs (German Landrace, female, 49 kg and 41 kg). In the ongoing in vivo study, all national and European laws, guidelines and policies for the care and use of laboratory animals are followed. The authors applied with the ARRIVE guidelines. The study was approved by the local authorities (Landesamt für Natur, Umwelt und Verbraucherschutz Nordrhein-Westfalen, aproval nr. 81-02.04.2020.A080). After initial placement, the cannulas were not readjusted during the experiment. The animals were deeply sedated to exclude spontaneous breathing. Nevertheless, regular sonographic controls were performed to exclude spontaneous diaphragmatic activity. Accordingly, all diaphragm activity was the result of PNS.

**Figure 2:**
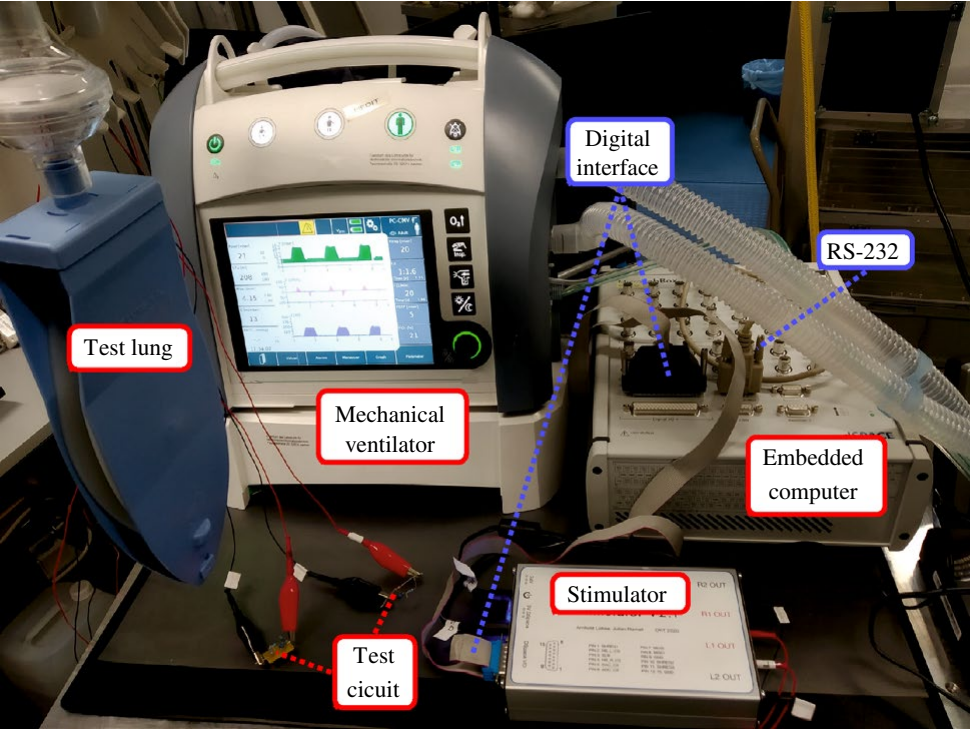
Technical setup in the laboratory during testing.

A modified mechanical ventilator (EVE IN, Fritz Stephan GmbH, Gackenbach, Germany) communicates with the embedded computer via a serial RS-232 interface. Through the RS-232 interface, the fraction of inspired oxygen (${F_{iO{_2}}}$), the mandatory respiratory frequency ($f_{mv}$), the positive end-expiratory pressure ($p_{PEEP}$), the inspiratory pressure ($p_{insp}$) and the time for a mandatory inspiration given by the mechanical ventilator $T_{mv,insp}$ can be adjusted via the embedded computer. The embedded computer receives the measured values of airway flow (${\dot{V}}$), airway pressure (*p*) and a ventilation phase (inspiration or expiration) signal from the mechanical ventilator. A sample time of ${10}\,ms$ was used. The test setup in the laboratory is shown in Fig. 2. During set up testing, a test lung and passive-resistive capacitive circuits replaced the study subjects.

### Stimulator design

**Figure 3:**
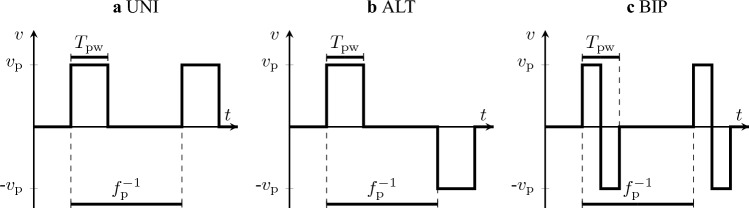
Settings and possible direction of a stimulation pulse.

The stimulator generates stimulation pulses to the phrenic nerve with a controlled voltage. The electric current applied to the stimulated tissue is limited by a simple internal resistance without additional monitoring circuitry. An overview of the investigated pulse settings is given in Fig. 3. Each pulse has a pulse voltage $\nu _{\textrm{p}}$, a pulse width $T_{\textrm{pw}}$ and is repeated with the pulse frequency $f_{\textrm{p}}$. The pulse direction can be either unidirectional (UNI), where the electrode polarity does not switch, alternating (ALT), where the electrode polarity switches after each pulse, or biphasic (BIP), where the electrode polarity changes within a pulse. To limit the number of possible combinations during PNS pattern evaluation, no further parameters such as a pause in between biphasic pulses or unbalanced biphasic pulses were introduced.

**Figure 4:**
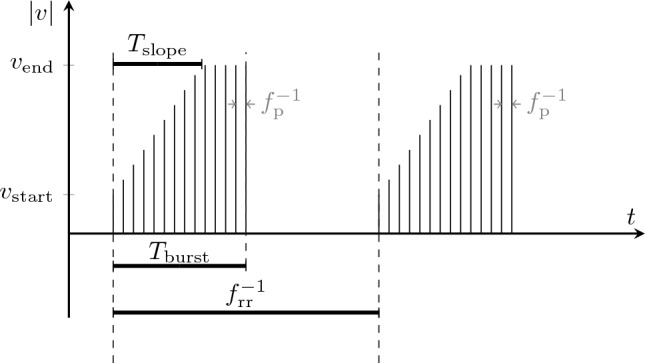
Burst settings for stimulation. The pulse frequency $f_{\textrm{p}}$ in gray shows the relation between pulses and bursts.

The embedded computer communicates with the stimulator to generate multiple pulses that form a stimulation bursts, as shown in Fig. 4. A burst should activate the diaphragm and cause a stimulated breath. Therefore, the burst frequency $f_{\textrm{rr}}$ is equal to the breathing rate of the PNS. Each burst is split into two phases. First, $\nu$ is increased from $\nu _{\textrm{start}}$ to $\nu _{\textrm{end}}$ over a duration of $T_{\textrm{slope}}$. This gradual increase is meant to induce a rather smooth than sudden contraction of the diaphragm. $\nu$ is then kept at $\nu _{\textrm{end}}$ for the remaining time of the burst duration $T_{\textrm{burst}}$.

**Figure 5:**
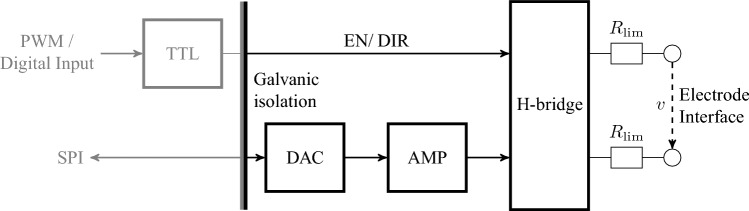
Functional diagram of the stimulator. EN and DIR denote the ENABLE- and DIRECTION-signal, respectively. $R_{{\textrm{lim}}}$ denotes the current limiting resistors.

An early design of the stimulator was previously presented in 2021^11^, but was modified since then and first used in 2022^12^. The functional diagram is shown in Fig. 5. The stimulator can be programmed externally via digital outputs and SPI. For safety, the maximum voltage of $\nu _{\textrm{p}}$ was set to 20 V, which leads in combination to the limiting resistors to a maximum stimulation current of approximately 12.0 mA.

Further, to ensure electrical safety a medical grade power supply (AKM65US24C2, XP Power, Pangbourne, United Kingdom) is used, and all signals are transferred through a medical grade galvanic isolator (ADuM2400/ ADuM2401, Analog Devices, Inc., Wilmington, U.S.A.). Through SPI, a digital-to-analog converter (DAC) (DAC80508, Texas Instruments Incorporated, Dallas, U.S.A.) generates an analog voltage between 0 and 5*V*, which is amplified through an operational amplifier (LM324, Texas Instruments Incorporated), leading to the pulse voltage $\nu _{\textrm{p}}$ between 0 and 20*V*. $\nu _{\textrm{p}}$ is routed through the H-bridge designed with analog switches (DG412FDY, Maxim Integrated, San José, U.S.A.) to either the first or second output port depending on the logic level of the ENABLE-and DIRECTION signal, while the other output port is connected to the electrical reference. The ENABLE-and DIRECTION signals are generated by pulse width modulation (PWM) and static digital signals sent by the embedded computer, which are processed in a transistor-transistor-logic (TTL) circuit with logic gates and flip-flops.

In both animal tests, the used hardware was identical with one exception: in the first animal test, a single stimulator with two electrode pairs, one for each phrenic nerve, was used. The hardware was changed to enable future individual stimulation with two galvanically isolated stimulators.

### Automated PNS pattern evaluation

For the evaluation of PNS patterns, the definition of the parameter ranges of the pulse settings $T_{\textrm{pw}}$ and $f_{\textrm{p}}$ is crucial. The parameter ranges which were reported in other studies using PNS are summarized in Table 1. To further specify the range of $T_{\textrm{pw}}$, we measured prior to the PNS pattern evaluation the strength-duration curve in our experiments. The strength-duration curve proposed by Lapicque in 1907^13^ shows the relation between the minimum required stimulation amplitude to excite a nerve and $T_{\textrm{pw}}$. During the measurement, $f_{\textrm{p}}$ was set to 100 Hz. The phrenic nerve was considered excited if the maximum ${\dot{V}}$ of the PNS burst was larger than 0.05 L/s.

The total parameter range of the evaluated PNS patterns is shown in Table 2. Because the stimulator is powered by an external power supply, there is no technical limitation on the electrical energy used in a stimulation pulse. To limit the parameter space, the maximum $f_{\textrm{p}}$ was set to ${208}\,\textrm{Hz}$, which is already twice as high as the maximum $f_{\textrm{p}}$ in Table 1. PNS patterns with $f_{\textrm{p}}$ below ${10}\,Hz$ and PNS patterns with unipolar pulses were found to be not suitable in initial tests and were omitted in the final PNS evaluation.

**Figure 6:**
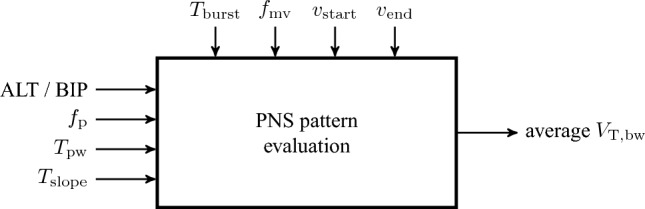
Overview of varying (left) and fixed (top) parameters during PNS pattern evaluation.

Two settings of $T_{\textrm{slope}}$ were chosen to limit the number of evaluated PNS parameter combinations. The parameters $T_{\textrm{burst}}$ and $f_{\textrm{rr}}$ were fixed as they do not change the overall shape of a PNS pattern, but rather have similar functions to the mechanical ventilator settings $T_{\textrm{mv,insp}}$ and $f_{mv}$, respectively. The parameters were set to provide an adequate gas exchange. Figure 6 gives an overview of variable and fixed parameters during PNS pattern evaluation.

Since the pulse direction and the time slope $T_{\textrm{slope}}$ have each two different settings, the PNS patterns were divided into the four subgroups {ALT, $T_{\textrm{slope}}$= 0.5 s}, {ALT, $T_{\textrm{slope}}$= 1.0 s}, {BIP, $T_{\textrm{slope}}$= 0.5 s} and {BIP, $T_{\textrm{slope}}$= 1.0 s}. The subgroup {ALT, $T_{\textrm{slope}}$ = 1.0 s} consists of 71 PNS patterns, while the other subgroups consist of 70 PNS patterns each.

To capture time-dependent effects during the evaluation, we used a baseline PNS pattern. The voltages $\nu _{\textrm{start}}$ and $\nu _{\textrm{end}}$ were set such that the baseline pattern ($T_{\textrm{pw}} = {4}\,\textrm{ms}$, $f_{\textrm{p}} = {200}\,\textrm{Hz}$, $T_{\textrm{slope}} = {1.0}$ s, ALT (I) / BIP (II)) achieved body weight scaled tidal volumes $V_{\textrm{T,bw}}$ between 5 and 6.5 mL/kg initially. The baseline PNS patterns were chosen after a short initial empirical trial.

**Figure 7:**
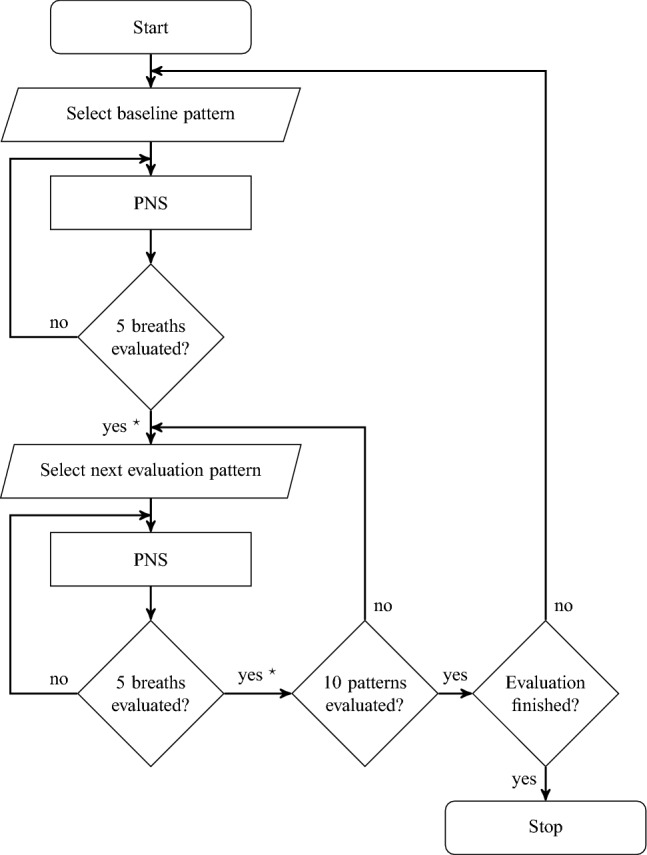
Program sequence to evaluate PNS patterns automatically. $^\star$ denotes the calculation of $V_{\textrm{T,bw}}$ according to Eq. 1.

The evaluation of PNS patterns was automated by the program sequence shown in Fig. 7. We started each evaluation with the baseline PNS pattern for five breaths. For each breath, $V_{\textrm{T,bw}}$ was calculated with

1 $$\begin{aligned} V_{\textrm{T,bw}} = \max _{T \in [0,T_{\textrm{breath}}]} \int _{t_{\textrm{k}}}^{t_{\textrm{k}}+T} \frac{1}{m_{\textrm{body}}}\ {\dot{V}}(\tau ) d\tau , \end{aligned}$$

where $T_{{\textrm{breath}}}$ denotes the time of one breath and $m_{\textrm{body}}$ the body weight. The mean $V_{\textrm{T,bw}}$ over all breaths quantifies the effectiveness of the baseline PNS pattern, then the first PNS pattern is tested by stimulation for five breaths. For this PNS pattern, $V_{\textrm{T,bw}}$ is calculated the same as before and the next PNS pattern is evaluated. After every ten evaluated PNS patterns, the baseline PNS pattern is evaluated again to capture changes of the general PNS effectiveness over time. The cycle between the evaluation of the baseline PNS pattern and untested PNS patterns continues until all PNS patterns were tested.

**Figure 8:**
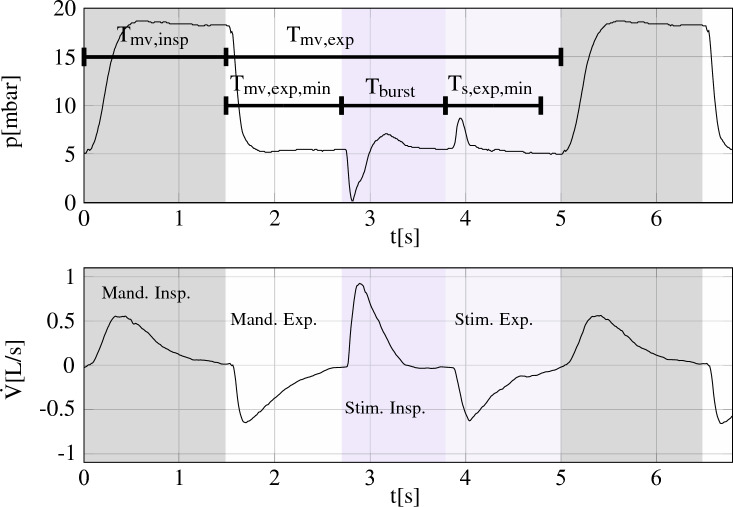
Timing of PNS breaths between mechanical ventilated breaths. $T_{\textrm{mv,insp}}$ and $T_{\textrm{mv,exp}}$ are given by the mechanical ventilator’s ventilatory phase signal. The curves were recorded during our experiments. Gray and white mark mandatory inspiration and expiration, respectively, while blue and light blue mark stimulated inspiration and expiration.

The mechanical ventilator is set to low frequency pressure controlled mandatory ventilation to ensure an adequate gas exchange. The software on the embedded computer times the PNS bursts, such that they are placed in between mechanically ventilated breaths, as shown in Fig. 8. During our experiment, the mechanical ventilator was set to $f_{mv}=f_{\textrm{rr}}$ = 13 (I)/12/min (II) and $T_{\textrm{mv,insp}}$=1.4 (I)/1.5s (II). $f_{\textrm{mv}}$, $T_{\textrm{mv,insp}}$ and $T_{\textrm{mv,exp}}$ are known to the embedded computer, the user inputs are $T_{\textrm{burst}}$, the minimum time for mandatory expiration $T_{\textrm{mv,exp,min}}$ and for PNS expiration $T_{\textrm{s,exp,min}}$. We made sure that no air trapping occurred during the experiments.

## Results

**Figure 9:**
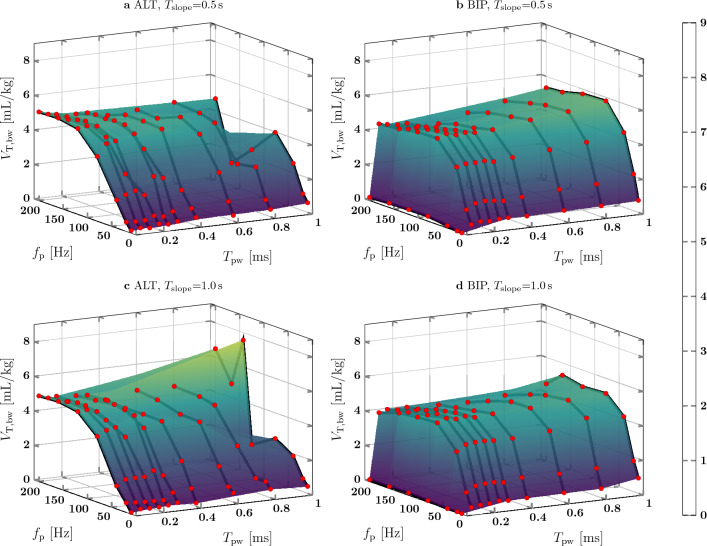
PNS pattern evaluation from animal test I.

In both animal tests, $V_{\textrm{T,bw}}$ of the baseline PNS pattern did not show an increasing or decreasing trend over time (I: $\mu =\mathrm{4.9\,mL/kg}, \sigma =\mathrm{0.30\,mL/kg}$, II: $\mu =\mathrm{6.4\,mL/kg}, \sigma =\mathrm{0.70\,mL/kg}$). In Fig. 9 for animal test I, $V_{\textrm{T,bw}}$ for different combinations of $f_{\textrm{p}}$ and $T_{\textrm{pw}}$ are plotted as markers and an interpolating surface is fit over all data points. The graph shape for $T_{\textrm{slope}}$ = 0.5 s and $T_{\textrm{slope}}$ = 1.0 s is similar for both ALT and BIP pulses. For PNS patterns with ALT pulses, $V_{\textrm{T,bw}}$ increased up to 5.2 mL/kg for an increasing $f_{\textrm{p}}$ between 10 Hz and approx. 120 Hz. A further increase of $f_{\textrm{p}}$ did not always lead to an increased $V_{\textrm{T,bw}}$. For e.g. $T_{\textrm{slope}}$ = 0.5 s and $T_{\textrm{pw}}$ = 0.1 ms, $V_{\textrm{T,bw}}$ was 4.9 mL/kg at $f_{\textrm{p}}$ = 120 Hz and 4.8 mL/kg at $f_{\textrm{p}}$ = 200 Hz. PNS patterns with BIP pulses had an increasing $V_{\textrm{T,bw}}$ for an increasing $f_{\textrm{p}}$ until a plateau was reached at approx. 80 Hz. While $V_{\textrm{T,bw}}$ was almost zero for $T_{\textrm{pw}}$= 0.05 ms, the plateau of $V_{\textrm{T,bw}}$ was reached at $T_{\textrm{pw}}$= 0.15 ms. For $f_{\textrm{p}}$ = 80 Hz and $T_{\textrm{slope}}$ = 0.5s, an increase of $T_{\textrm{pw}}$ from 0.15 to 1.0 ms lead to an increase of $V_{\textrm{T,bw}}$ from 4.9 to 5.8 mL/kg. Regarding the PNS patterns with $T_{\textrm{slope}}$ = 1.0s, $V_{\textrm{T,bw}}$ increased from 4.7 to 5.1 mL/kg. In the subgroup {ALT, $T_{\textrm{slope}}$= 1.0s}, there were two PNS patterns ($f_{\textrm{p}}$ $\in [142, 200]~Hz$, $T_{\textrm{pw}}$ = 1.0ms) which generated a $V_{\textrm{T,bw}}$ up to 8.2 mL/kg.

**Figure 10:**
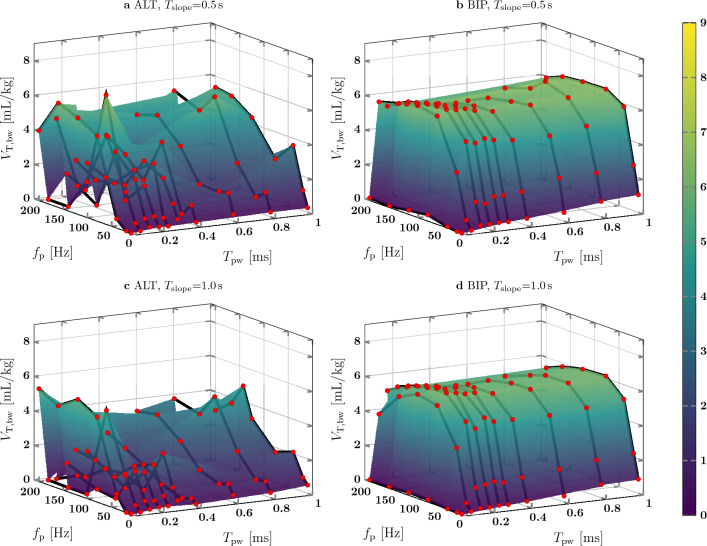
PNS pattern evaluation from animal test II.

The results of the PNS pattern evaluation of animal test II are shown in Fig. 10. The $V_{\textrm{T,bw}}$ surface shape for ALT pulses is different compared to animal test I and no clear shape is detectable. For BIP pulses, the $V_{\textrm{T,bw}}$ surface shape is similar to the shape of animal test I. $V_{\textrm{T,bw}}$ increased with $f_{\textrm{p}}$ up to 80 Hz for $T_{\textrm{pw}}$ greater or equal to 0.1 ms and increased with $T_{\textrm{pw}}$ up to 0.15ms. For $f_{\textrm{p}}$ = 80 Hz and $T_{\textrm{slope}}$ = 0.5 s, $V_{\textrm{T,bw}}$ increased from 6.3 mL/kg at $T_{\textrm{pw}}$ = 0.15ms to 6.5 mL/kg at $T_{\textrm{pw}}$ = 1.0 ms. However, for the PNS patterns with $T_{\textrm{slope}}$ = 1.0 s, $V_{\textrm{T,bw}}$ decreased from 6.2 to 6.1 mL/kg.

**Figure 11:**
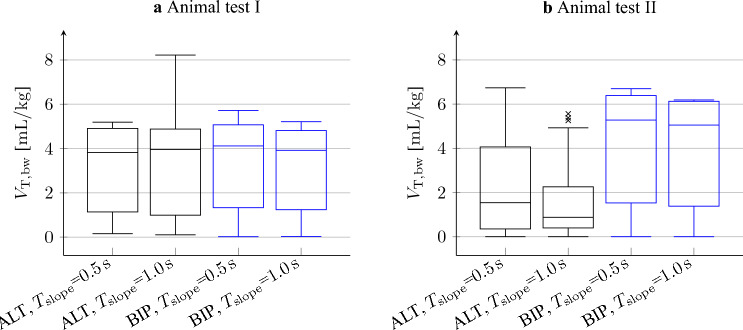
Box plot of the PNS pattern evaluation. The patterns were divided into subgroups by pulse directions and $T_{\textrm{slope}}$. The boxes indicate the lower and higher quartile. The bar denotes the median. Lower and upper whisker mark the minimum and maximum of $V_{\textrm{T,bw}}$, disregarding outliers. Outliers marked by “x” were detected if $V_{\textrm{T,bw}}$ lied farther than 1.5 interquartile ranges above the upper or below the lower quartile.

Medians and standard deviations of $V_{\textrm{T,bw}}$ during PNS pattern evaluation of both animal tests are shown in Fig. 11. In animal test I, the median and interquartile ranges of all PNS pattern subgroups are similar. The minimum and maximum median are 3.82 mL/kg and 4.12 mL/kg, respectively. The interquartile ranges lie in between 3.57 and 3.89 mL/kg and are therefore almost as big as the median. During animal test II in PNS patterns with ALT pulses, the median and upper quartile $V_{\textrm{T,bw}}$ were lower than in BIP pulses (Median ALT: 0.9–1.5 mL/kg,BIP: 5.0–5.3 mL/kg; Upper quartile ALT: 2.3–4.1 mL/kg, BIP: 6.1–6.4 mL/kg). Except for ALT pulses in animal test II, the median and interquartile range are similar for $T_{\textrm{slope}}$ = 0.5 s and $T_{\textrm{slope}}$ = 1.0 s.

To quantify the overall effect of $f_{\textrm{p}}$ and $T_{\textrm{pw}}$, we investigated the respective correlation coefficients between them and $V_{\textrm{T,bw}}$. The correlation coefficients between $f_{\textrm{p}}$ and $V_{\textrm{T,bw}}$ for both animal tests are shown in Table 3. For PNS patterns with ALT pulses, correlation coefficients between animal test I and II differ, while the correlation is similar in both animal tests for PNS patterns with BIP pulses. The correlation coefficients between PNS patterns with $T_{\textrm{slope}}$ equal to 0.5 s and 1.0 s are similar.

The correlation coefficients between $T_{\textrm{pw}}$ and $V_{\textrm{T,bw}}$ for both animal tests are shown in Table 4. Similar to before, the correlation coefficients between the animal tests differ for PNS patterns with ALT pulses, but are similar for PNS patterns with BIP pulses. In general, the correlation coefficients are lower than the correlation coefficients between $f_{\textrm{p}}$ and $V_{\textrm{T,bw}}$. It should be noted that the *p*-values are higher than 0.001.

## Discussion

Our goal was to evaluate the influence of PNS parameters on PNS effectivity, which we quantified with $V_{\textrm{T,bw}}$. In Section “Results”, based on a few pilot trials, a systematic evaluation of PNS patterns has been presented. The box plot in Fig. 11 shows a large interquartile range compared to the median in all subgroups of the PNS patterns in both animal tests. The interquartile range indicates a high variability in between patterns, which leads to the assumption that $V_{\textrm{T,bw}}$ depends on $T_{\textrm{pw}}$ and $f_{\textrm{p}}$. This assumption is supported by the fact that both parameters determine the amount of electrical energy of pulses and bursts to excite the phrenic nerve.

In Figs. 9 and 10, the surface shape of $V_{\textrm{T,bw}}$ depending on $f_{\textrm{p}}$ and $T_{\textrm{pw}}$ is different in between animal tests for ALT pulses, but is similar for BIP pulses. The correlation coefficients between $V_{\textrm{T,bw}}$ and $f_{\textrm{p}}$ given in Table 3 are different for ALT pulses in between animal tests (I:0.84–0.87, II: 0.42–0.51), but are similar for BIP pulses (I: 0.55–0.57, II: 0.56–0.58). Therefore, PNS patterns with BIP pulses seem to achieve generally a more consistent $V_{\textrm{T,bw}}$ than PNS with ALT pulses.

Table. 3 shows a high correlation between $f_{\textrm{p}}$ and $V_{\textrm{T,bw}}$. Phrenic nerve excitation leads to the excitation of smaller diaphragm motor units that then excite larger diaphragm motor units with increasing $f_{\textrm{p}}$^14^, p. 81. Except for the PNS pattern evaluation of ALT pulses in animal test II and a few outliers, $V_{\textrm{T,bw}}$ increased with $f_{\textrm{p}}$ until a threshold value of approximately 120 Hz for ALT pulses and 80 Hz for BIP pulses was reached. This behavior leads to the assumption that with these PNS parameters, all large diaphragm motor units were excited. Since most referenced literature^6,7,15^ used $f_{\textrm{p}}$ between 5 and 60 Hz, it may have been possible to generate a higher $V_{\textrm{T,bw}}$ with an increased $f_{\textrm{p}}$. However, Günter et al.^16^ summarized in their review of safe long-term stimulation parameters, that in PNS with low frequencies, such as 36 Hz^17^, the neural damage was found to be minimal, whereas PNS with high frequencies, such as 50 Hz and 100 Hz^18^, were reported to cause neural damage in cats. It should be noted that the cats had implanted electrodes and received stimulation amplitudes twice as high as required for full recruitment. Therefore, we recommend for a given pulse amplitude to increase $f_{\textrm{p}}$ only up to the threshold value at which the plateau is reached. In our experiments, the plateau may have been reached for $f_{\textrm{p}}$ below 50 Hz if we had set higher pulse amplitudes.

**Figure 12:**
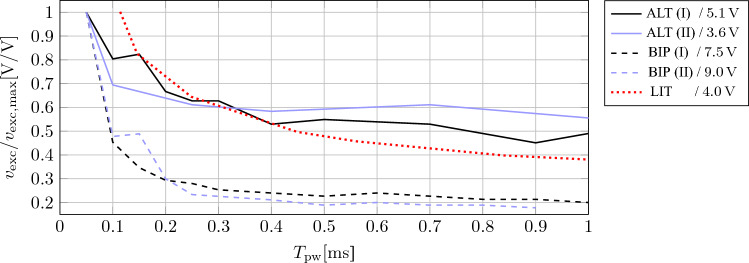
Strength-duration curve of the phrenic nerve for ALT and BIP pulses compared to literature (LIT, Fig. 1 from Huizar et al.^19^). The roman numbers denote the first (I) and second (II) animal test, respectively. For better comparison between experiments, the phrenic nerve excitation voltage $\nu _{\textrm{exc}}$ was divided by the maximum excitation voltage $v_{\textrm{exc,max}}$ of the respective experiment. $v_{\textrm{exc,max}}$ is given in the legend for each experiment.

BIP pulse PNS patterns with $T_{\textrm{pw}}$ of 0.05 ms were ineffective, possibly because the low pulse energy did not excite a neuron which was depolarized after the pulse direction was switched. However, increasing $T_{\textrm{pw}}$ above 0.15 ms did not always lead to an increase of $V_{\textrm{T,bw}}$. This finding is in accordance to the strength-duration curves from our experiments and from reported literature^19^, as shown in Fig. 12. The stimulation strength to excite the phrenic nerve in our experiment barely decreases for $T_{\textrm{pw}}$ above approximately 0.2 ms. Therefore, we assume that for the given voltage, a higher $T_{\textrm{pw}}$ cannot excite more phrenic nerve fibers. According to Günter et al.^16^, $T_{\textrm{pw}}$ should be kept short to minimize electrode corrosion. These findings and arguments support the decision of the authors of the referenced literature^6,7,10,15^ to use BIP pulses. However, since the ALT pulse PNS patterns with $T_{\textrm{pw}}$ = 0.05 ms generated similar results in both animal tests, these PNS patterns are also considered suitable for PNS since $T_{\textrm{pw}}$ is low.

For $T_{\textrm{slope}}$ = 0.5 s and $T_{\textrm{slope}}$ = 1.0 s, the overall shape of Figs. 9 and 10 is similar. In addition, the results of the box plot in Fig. 11 and the correlation coefficients given in Tables 3 and 4 are close to each other. Therefore, $T_{\textrm{slope}}$ has a weak effect on $V_{\textrm{T,bw}}$, but it helps to achieve a smooth diaphragm contraction which may be better tolerated by a patient. A single ALT pulse PNS pattern ($f_{\textrm{p}}$ = 142 Hz, $T_{\textrm{pw}}$ = 1.0 ms) generated a remarkably higher $V_{\textrm{T,bw}}$ than PNS patterns with similar $f_{\textrm{p}}$ and $T_{\textrm{pw}}$ settings in both animal tests. Further investigation would be necessary to find the underlying reason.

Regarding limitations, our study does not include an analysis of the start voltage $\nu _{\textrm{start}}$ and the end voltage $\nu _{\textrm{end}}$ of the PNS burst, which will affect $V_{\textrm{T,bw}}$. Further, $V_{\textrm{T,bw}}$ does not only dependent on voltage and the PNS pattern, but also on the anatomy and location of the electrodes. Since the stimulator hardware changed (galvanic isolation between the electrode interface of the left and right phrenic nerve) in between both animal tests, the generated $V_{\textrm{T,bw}}$ between animal test I and II are not directly comparable. Even though the baseline PNS patterns in both animal tests did not show time-variant effects, there may be time-varying effects that affect other PNS patterns. Further, all PNS patterns were tested for a duration of five breaths. Since the PNS pattern evaluation was conducted with voltage-controlled pulse stimulation, it remains unclear if the findings are the same for current-controlled pulses. Therefore, more experiments may be required to validate the proposed guideline of our pilot study.

## Conclusion

PNS is capable of keeping the diaphragm conditioned and may avoid diaphragm atrophy. Our paper shows the necessary technical setup, the coordination between PNS and mechanical ventilation and an automatic pattern switching algorithm to systematically evaluate different PNS patterns. The methods were applied in a pilot study.

We concluded the following guideline for the selection of PNS patterns: the effect of $T_{\textrm{slope}}$ on $V_{\textrm{T,bw}}$ is weak, but it helps to achieve a smooth diaphragm contraction. BIP pulse PNS was more consistent than ALT pulse PNS, but ALT pulse PNS with a short $T_{\textrm{pw}}$ can also be effective. For BIP pulse PNS, $T_{\textrm{pw}}$ and $f_{\textrm{p}}$ have saturation limits, at which $V_{\textrm{T,bw}}$ does not or only minimally increases. Therefore, to minimize the risk of electrode corrosion and to minimize the amount of electrical energy during PNS, both should be increased until the saturation of $V_{\textrm{T,bw}}$ is reached. The guideline is helpful for future clinical applications of phrenic nerve stimulation.

**Table 1:** Parameter ranges reported in other PNS studies.

Electrode placement	Study	$T_{\textrm{pw}}$ (ms)	$f_{\textrm{p}}$ (Hz)	References
Percutaneous	Human	0.15–0.30	5–25	^15^
Transcutaneous	Human	0.01–0.40	10–100	^10^
Transvenous	Pig	0.07–0.30	40–60	^6^
Transvenous	Human	0.10–0.30	–	^7^

In the studies, biphasic current-controlled pulses were used and the electrode placement method is different.

**Table 2:** PNS parameter ranges of the evaluation.

Parameter	Description	Value range	Unit	Fix
$T_{\textrm{pw}}$	Pulse width	0.05–1.0	ms	No
$f_{\textrm{p}}$	Pulse frequency	10–208	Hz	No
DIR	Pulse direction	ALT/BIP		No
$T_{\textrm{slope}}$	Time slope in which the voltage increases from start to end voltage	0.5/1.0	s	No
$T_{\textrm{burst}}$	Duration of one burst	1.10	s	Yes
$f_{\textrm{rr}}$	Duration of one burst	13 (I)/12 (II)	$1/\textrm{min}$	Yes
$\nu _{\textrm{start}}$	Start voltage of stimulation burst (left)	6.50 (I)/7.50 (II)	V	Yes
$\nu _{\textrm{start}}$	Start voltage of stimulation burst (right)	6.50 (I)/7.50 (II)	V	Yes
$\nu _{\textrm{end}}$	End voltage of stimulation burst (left)	10.0 (I)/10.0 (II)	V	Yes
$\nu _{\textrm{end}}$	End voltage of stimulation burst (right)	10.0 (I)/12.0 (II)	V	Yes

**Table 3:** Pearson correlation coefficients (two-tailed) between $V_{\textrm{T,bw}}$ and $f_{\textrm{p}}$ for animal test I and II.

$f_{\textrm{p}}$	ALT (I)	ALT (II)	BIP (I)	BIP (II)
$T_{\textrm{slope}} = {0.5}s$	0.84	0.51	0.57	0.58
$T_{\textrm{slope}} = {1.0}s$	0.87	0.42	0.55	0.56

The *p*-values of all coefficients are below 0.001.

**Table 4:** Pearson correlation coefficients (two-tailed) between $V_{\textrm{T,bw}}$ and $T_{\textrm{pw}}$ of animal test I and II.

$T_{\textrm{pw}}$	ALT (I)	ALT (II)	BIP (I)	BIP (II)
$T_{\textrm{slope}} = {0.5}s$	− 0.10 (0.428)	0.21 (0.079)	0.32 (0.008)	0.27 (0.020)
$T_{\textrm{slope}} = {1.0}s$	− 0.02 (0.856)	0.22 (0.066)	0.28 (0.019)	0.25 (0.034)

The *p*-values are shown in brackets.

## Data Availability

The datasets used and analyzed during the current study are available from the corresponding author on reasonable request.
